# Supplementary material for: A Likelihood Approach for Real-Time Calibration of Stochastic Compartmental Epidemic Models
Source: PLoS Comput Biol. 2017 Jan 17;13(1):e1005257. doi: 10.1371/journal.pcbi.1005257 (PMC5240920; doi:10.1371/journal.pcbi.1005257)
Supplement: S1 File — (TAR.GZ) [file pcbi.1005257.s014.tar.gz › HSPH_Online-SI-Revision/PF08/n10-severe/n10-severe_PF_table0.pdf]

1	R0	{1.68952, 11.8402}	{1.8032, 19.6887}	{{1.71265, 19.2112}, {1.91535, 20.}}	{7.21702, 66.2873}	inf	1046.	1359.3	{1261.39, 1502.37}	31.071	0.117164	newly	589.	472.66	{437., 521.}	19.7521	0.0968421	Reff	0.898487	0.946336	{0.864798, 1.01361}	7.01832	ar	6872.	8632.95	{8355., 9043.}	25.625
2	R0	{1.71739, 9.62661}	{2.00486, 19.8854}	{{1.85606, 20.}, {2.1532, 20.}}	{17.083, 106.567}	inf	945.	1690.11	{1540.93, 1802.32}	78.8477	0.250719	newly	711.	593.87	{539., 637.}	16.474	0.0791314	Reff	0.984408	1.0367	{0.953367, 1.11778}	6.75068	ar	6874.	9309.42	{9030., 9617.}	35.4294
3	R0	{1.48936, 6.23975}	{2.0554, 19.1779}	{{1.81681, 16.7795}, {2.2307, 20.}}	{38.0058, 207.35}	inf	625.	1816.65	{1473.53, 2077.44}	190.664	0.459247	newly	640.	660.45	{595., 741.}	7.45156	0.0315569	Reff	0.888701	1.04031	{0.934808, 1.13558}	17.1576	ar	5666.	9225.6	{8610., 9553.}	62.8239
4	R0	{1.62394, 7.51135}	{1.72064, 19.7799}	{{1.66089, 19.4119}, {1.75616, 20.}}	{6.28781, 163.334}	inf	753.	1266.97	{1126.18, 1329.7}	68.2564	0.224595	newly	670.	437.94	{396., 468.}	34.6358	0.185602	Reff	0.945133	0.944789	{0.911674, 0.972951}	2.36239	ar	6417.	8329.86	{8147., 8512.}	29.8093
5	R0	{1.53632, 8.57652}	{1.7212, 19.8745}	{{1.62523, 19.3696}, {1.82595, 20.}}	{12.0452, 131.732}	inf	707.	1278.86	{1082.81, 1385.41}	80.8858	0.255091	newly	572.	442.35	{373., 488.}	22.6661	0.114088	Reff	0.929627	0.954183	{0.901497, 0.992706}	4.60795	ar	6260.	8455.28	{8015., 8776.}	35.0684
6	R0	{1.47166, 5.7434}	{1.73852, 19.8402}	{{1.67487, 19.4422}, {1.83642, 20.}}	{18.1331, 245.443}	inf	531.	1234.68	{1169.54, 1292.79}	132.52	0.366009	newly	615.	431.94	{398., 462.}	29.7659	0.154303	Reff	0.960847	1.02132	{0.980032, 1.0546}	6.51725	ar	5650.	8243.57	{8013., 8478.}	45.9039
7	R0	{1.48179, 8.87369}	{1.69709, 19.7012}	{{1.60637, 19.3848}, {1.84555, 20.}}	{14.5297, 122.018}	inf	480.	1171.56	{974.028, 1352.01}	144.075	0.383551	newly	360.	407.42	{346., 462.}	14.5444	0.0572934	Reff	0.902706	0.940303	{0.880616, 0.995784}	5.58668	ar	5233.	8243.09	{7881., 8751.}	57.5213
8	R0	{1.53669, 6.47807}	{2.15047, 19.8807}	{{2.01869, 20.}, {2.22647, 20.}}	{39.9417, 206.893}	inf	675.	1866.15	{1704.14, 2056.43}	176.467	0.440076	newly	686.	655.7	{598., 717.}	6.79883	0.0307943	Reff	0.902344	1.04064	{0.976547, 1.10193}	15.3309	ar	5934.	9527.41	{9321., 9684.}	60.5563
9	R0	{1.57227, 6.20989}	{2.34362, 18.442}	{{1.97642, 13.8796}, {2.66237, 20.}}	{49.0599, 196.978}	inf	741.	2165.39	{1550.93, 2694.31}	192.225	0.452399	newly	787.	844.3	{716., 986.}	12.028	0.0491225	Reff	1.05279	1.25069	{1.10465, 1.34004}	18.9382	ar	6136.	9454.6	{8790., 10 004.}	54.0841
10	R0	{1.54936, 6.50938}	{2.36499, 17.9343}	{{2.06836, 15.0965}, {2.69692, 20.}}	{52.6432, 175.514}	inf	717.	2255.96	{1887.98, 2754.99}	214.639	0.490943	newly	740.	862.57	{765., 967.}	17.0122	0.0666009	Reff	0.879107	0.953966	{0.898611, 1.01277}	9.25856	ar	6275.	9523.02	{8960., 9952.}	51.7613
11	R0	{1.42179, 3.18601}	{2.34566, 19.7031}	{{2.20639, 18.9369}, {2.47992, 20.}}	{64.9792, 518.427}	inf	449.	2377.17	{1978.88, 2612.87}	429.437	0.721364	newly	893.	857.12	{774., 941.}	6.79283	0.0306791	Reff	0.921746	1.16132	{1.08628, 1.21466}	25.9917	ar	5322.	9892.4	{9575., 10 158.}	85.8775
12	R0	{1.48035, 6.46183}	{2.1808, 18.7016}	{{2.06637, 17.1556}, {2.33332, 20.}}	{47.3168, 189.416}	inf	718.	1984.92	{1818.55, 2236.92}	176.452	0.438267	newly	750.	769.26	{684., 833.}	7.64533	0.0330992	Reff	0.99065	1.25701	{1.1719, 1.32763}	26.887	ar	5793.	9807.06	{9457., 10 106.}	69.2916
13	R0	{1.75755, 8.53854}	{2.16579, 19.9316}	{{2.01422, 20.}, {2.22303, 20.}}	{23.228, 133.431}	inf	1074.	1841.07	{1734.44, 1945.89}	71.4217	0.233343	newly	871.	644.91	{603., 679.}	25.9575	0.131115	Reff	0.980361	1.04338	{0.962199, 1.09619}	7.08484	ar	6832.	9557.94	{9319., 9769.}	39.8996
14	R0	{1.47355, 4.89451}	{2.20189, 18.5547}	{{2.02502, 16.4075}, {2.41295, 20.}}	{49.4274, 279.093}	inf	528.	2118.79	{1843.31, 2465.57}	301.286	0.600033	newly	648.	788.37	{705., 847.}	21.8133	0.0847544	Reff	0.862321	0.986569	{0.906816, 1.0485}	14.559	ar	5710.	9477.99	{9065., 9858.}	65.9893
15	R0	{1.62071, 5.98665}	{2.76198, 18.3176}	{{2.33085, 14.8881}, {3., 20.}}	{70.4177, 205.974}	inf	906.	3010.76	{2343.18, 3512.43}	232.313	0.51714	newly	967.	1144.44	{1059., 1256.}	18.5336	0.0729997	Reff	0.893659	1.09205	{1.0006, 1.19502}	22.2918	ar	6635.	10 513.5	{10 014., 10 758.}	58.4555
16	R0	{1.67268, 7.7256}	{2.26435, 17.9103}	{{1.76991, 10.9483}, {2.51875, 20.}}	{35.3727, 131.83}	inf	927.	2200.43	{1304.26, 2652.31}	137.371	0.361661	newly	767.	822.03	{728., 893.}	9.20078	0.0379737	Reff	0.766087	0.857514	{0.783642, 0.923068}	12.5379	ar	6947.	9801.94	{8586., 10 277.}	41.096
17	R0	{1.67805, 11.1417}	{2.03454, 19.206}	{{1.75181, 15.3266}, {2.17682, 20.}}	{21.2727, 72.3792}	inf	929.	1620.94	{1160.14, 1884.14}	74.4818	0.23604	newly	581.	602.	{522., 672.}	8.73322	0.0370549	Reff	1.07479	1.18729	{1.10831, 1.26581}	10.5714	ar	6625.	9058.93	{8208., 9401.}	36.7386
18	R0	{1.66743, 9.99046}	{2.01435, 19.4828}	{{1.78144, 17.1724}, {2.18577, 20.}}	{21.1071, 95.0139}	inf	904.	1597.17	{1331.59, 1794.78}	76.8527	0.242977	newly	639.	571.44	{492., 630.}	10.6573	0.0507209	Reff	0.998457	1.06089	{1.00081, 1.12568}	6.66822	ar	6899.	8973.61	{8393., 9413.}	30.0712
19	R0	{1.70787, 9.85478}	{1.80294, 19.9715}	{{1.75001, 20.}, {1.85039, 20.}}	{5.62154, 102.658}	inf	940.	1422.09	{1363.75, 1479.95}	51.2857	0.179411	newly	617.	488.06	{460., 515.}	20.8979	0.102321	Reff	0.877333	0.925311	{0.889679, 0.955827}	5.46866	ar	6867.	8981.09	{8789., 9162.}	30.7862
20	R0	{1.53809, 10.5848}	{1.57287, 19.6525}	{{1.50503, 19.072}, {1.6403, 20.}}	{3.25562, 85.6668}	inf	660.	952.918	{855.325, 1089.81}	44.3815	0.157691	newly	432.	339.08	{301., 381.}	21.5139	0.107053	Reff	1.05928	1.0027	{0.97506, 1.02803}	5.34158	ar	6108.	7509.99	{7120., 7822.}	22.9533
21	R0	{1.59925, 6.74479}	{2.40243, 18.0562}	{{1.99557, 14.2843}, {2.78285, 20.}}	{50.222, 167.705}	inf	837.	2302.38	{1676.99, 2811.58}	175.076	0.43014	newly	789.	878.59	{776., 992.}	12.5057	0.049748	Reff	0.912212	1.01828	{0.949227, 1.10381}	11.9316	ar	6411.	9606.91	{8946., 10 128.}	49.8504
22	R0	{1.38779, 4.40977}	{1.87832, 18.3666}	{{1.76732, 15.7346}, {2.09312, 20.}}	{35.3463, 316.498}	inf	333.	1416.44	{1173.49, 1655.55}	325.358	0.625197	newly	507.	553.99	{500., 619.}	10.2623	0.0414114	Reff	0.993103	1.20251	{1.12003, 1.29885}	21.0865	ar	5154.	8696.88	{8318., 9266.}	68.7404
23	R0	{1.53657, 7.50352}	{1.95445, 18.5769}	{{1.7139, 14.8965}, {2.19043, 20.}}	{27.3118, 147.576}	inf	535.	1539.95	{1046.45, 1859.76}	187.841	0.448724	newly	479.	576.87	{487., 656.}	21.0251	0.0807662	Reff	0.991395	1.0645	{0.951161, 1.14097}	8.98166	ar	5928.	8826.31	{7939., 9411.}	48.8919
24	R0	{1.71131, 6.24526}	{2.19229, 19.183}	{{2.13161, 17.7617}, {2.26323, 20.}}	{28.1058, 207.161}	inf	964.	2131.07	{2020.5, 2252.35}	121.066	0.344109	newly	1016.	792.22	{738., 838.}	22.0256	0.108668	Reff	0.982634	1.14776	{1.05827, 1.21763}	16.8046	ar	6924.	9876.54	{9618., 10 084.}	42.6421
25	R0	{1.4412, 3.07729}	{2.74847, 19.8994}	{{2.68562, 20.}, {2.81464, 20.}}	{90.7073, 546.653}	inf	606.	2852.27	{2755.94, 2949.25}	370.672	0.672272	newly	1165.	1040.64	{982., 1092.}	10.6747	0.0493557	Reff	0.935195	1.31349	{1.25714, 1.39337}	40.4508	ar	5810.	10 351.5	{10 210., 10 485.}	78.1664
26	R0	{1.4159, 4.93858}	{1.99339, 15.5323}	{{1.59787, 9.97247}, {2.32004, 20.}}	{40.7862, 214.509}	inf	458.	1578.63	{961.109, 2101.58}	244.68	0.51766	newly	608.	724.88	{601., 836.}	20.3158	0.0781692	Reff	0.962104	1.15142	{1.05378, 1.28681}	19.6769	ar	5255.	8675.22	{7403., 9529.}	65.0851
27	R0	{1.57465, 7.05414}	{2.49, 18.836}	{{2.17375, 15.7172}, {2.7461, 20.}}	{58.1301, 167.02}	inf	797.	2295.01	{1867.81, 2724.14}	187.957	0.449495	newly	776.	884.7	{739., 994.}	16.1134	0.0638924	Reff	1.05486	1.31848	{1.15872, 1.45182}	24.991	ar	6359.	9690.22	{9283., 10 076.}	52.3859
28	R0	{1.41422, 4.62402}	{2.46995, 19.6522}	{{2.26223, 18.6504}, {2.75145, 20.}}	{74.6512, 325.002}	inf	570.	2490.89	{2050.91, 2960.32}	336.999	0.637046	newly	804.	910.52	{796., 1053.}	14.4602	0.0573767	Reff	0.944699	1.23436	{1.15471, 1.35752}	30.6619	ar	5485.	9975.93	{9654., 10 234.}	81.8766
29	R0	{1.41901, 3.63017}	{2.79943, 18.7446}	{{2.41795, 15.9712}, {3., 20.}}	{97.2808, 416.356}	inf	600.	3049.53	{2519.62, 3471.98}	408.255	0.703366	newly	1020.	1146.7	{1063., 1230.}	12.4471	0.0503151	Reff	0.854528	1.13622	{1.0448, 1.20698}	32.9646	ar	5979.	10 446.8	{10 082., 10 668.}	74.7245
30	R0	{1.60719, 4.72984}	{2.92606, 19.6113}	{{2.77855, 19.1341}, {3., 20.}}	{82.0607, 314.629}	inf	850.	3313.11	{3020.1, 3538.15}	289.778	0.588976	newly	1189.	1195.51	{1102., 1256.}	3.96552	0.017259	Reff	0.978457	1.20289	{1.09669, 1.30739}	22.937	ar	6479.	10 638.8	{10 457., 10 805.}	64.2048
31	R0	{1.38175, 6.26468}	{1.97476, 16.5558}	{{1.56741, 10.939}, {2.27769, 20.}}	{42.9173, 164.272}	inf	497.	1556.1	{921.461, 2052.22}	213.099	0.476397	newly	506.	650.54	{528., 754.}	28.7589	0.106071	Reff	0.889156	1.05733	{0.96536, 1.17375}	18.9137	ar	5397.	8631.43	{7410., 9459.}	59.9301
32	R0	{1.4947, 8.8847}	{1.62145, 19.5516}	{{1.53856, 19.0092}, {1.69247, 20.}}	{8.64424, 120.059}	inf	437.	1032.84	{872.698, 1175.01}	136.348	0.370247	newly	341.	362.61	{320., 414.}	9.27566	0.0380074	Reff	0.948387	0.930756	{0.874539, 0.997529}	3.90641	ar	5407.	7593.97	{7214., 7936.}	40.447
33	R0	{1.55891, 8.28978}	{1.67374, 19.8289}	{{1.58861, 19.4206}, {1.77463, 20.}}	{7.38866, 139.196}	inf	655.	1147.24	{967.028, 1299.78}	75.1512	0.241223	newly	521.	399.06	{348., 442.}	23.405	0.117866	Reff	0.96668	0.971252	{0.914014, 1.01274}	3.61935	ar	6239.	8085.2	{7681., 8440.}	29.5913
34	R0	{1.60764, 10.4729}	{1.71832, 19.6195}	{{1.63312, 19.3535}, {1.7993, 20.}}	{7.53102, 87.3355}	inf	772.	1241.23	{1088.89, 1349.4}	61.0094	0.203349	newly	469.	433.49	{383., 474.}	9.85714	0.0456367	Reff	0.879861	0.918147	{0.852308, 0.972662}	5.71736	ar	6495.	8341.56	{7961., 8805.}	28.4305
35	R0	{1.48527, 5.74485}	{2.17912, 18.502}	{{2.03399, 16.4915}, {2.34576, 20.}}	{46.7157, 222.062}	inf	650.	2008.59	{1765.12, 2354.28}	209.014	0.485281	newly	769.	771.68	{673., 843.}	6.6606	0.0291677	Reff	0.956217	1.16168	{1.08984, 1.24955}	21.4885	ar	5811.	9479.	{9175., 9883.}	63.1217
36	R0	{1.61457, 9.93364}	{1.87085, 19.3734}	{{1.66591, 15.5807}, {1.99223, 20.}}	{16.1859, 95.0284}	inf	799.	1466.18	{1136.07, 1732.82}	83.5023	0.258224	newly	557.	530.91	{462., 592.}	7.72531	0.0354591	Reff	1.02993	1.09219	{0.999669, 1.16335}	7.07773	ar	6338.	8891.49	{8247., 9394.}	40.2886
37	R0	{1.47831, 7.08139}	{2.00806, 18.5911}	{{1.73145, 15.4787}, {2.21542, 20.}}	{35.8351, 162.535}	inf	564.	1577.01	{1107.08, 1910.4}	179.611	0.437399	newly	542.	603.4	{521., 680.}	13.0554	0.0521779	Reff	0.993129	1.16802	{1.06001, 1.26853}	17.6494	ar	5530.	8989.37	{8292., 9492.}	62.5564
38	R0	{1.73445, 9.8858}	{2.14558, 19.6036}	{{2.01505, 17.4424}, {2.26992, 20.}}	{23.8767, 98.3008}	inf	986.	1967.61	{1778.02, 2257.7}	99.5549	0.296271	newly	672.	709.49	{628., 795.}	8.94792	0.0369643	Reff	1.0313	1.12916	{1.04967, 1.21459}	9.76192	ar	6842.	9464.2	{9293., 9728.}	38.3251
39	R0	{1.66621, 11.4824}	{1.80958, 19.363}	{{1.61413, 17.0022}, {1.94625, 20.}}	{10.2059, 68.632}	inf	863.	1388.29	{1056.99, 1610.78}	62.1796	0.205462	newly	517.	496.76	{418., 564.}	9.04836	0.0415731	Reff	1.00256	1.01791	{0.935209, 1.08256}	5.06205	ar	6473.	8692.29	{7860., 9116.}	34.2853
40	R0	{1.60694, 9.95741}	{1.84021, 19.7186}	{{1.7045, 19.9716}, {1.91789, 20.}}	{14.8123, 98.0293}	inf	852.	1491.95	{1304.98, 1656.42}	75.2556	0.240191	newly	560.	525.73	{451., 588.}	8.2375	0.0384597	Reff	0.960147	1.01388	{0.964918, 1.04608}	6.00576	ar	6625.	8828.45	{8455., 9131.}	33.2596
41	R0	{1.55128, 4.71911}	{2.68825, 19.0468}	{{2.28549, 15.22}, {3., 20.}}	{73.2926, 303.611}	inf	710.	2760.48	{2142.79, 3210.23}	288.8	0.585181	newly	1008.	1039.5	{957., 1141.}	5.90873	0.024867	Reff	1.00632	1.24716	{1.1632, 1.33665}	23.9329	ar	6141.	10 038.5	{9609., 10 330.}	63.4675
42	R0	{1.60063, 6.81118}	{1.85263, 19.96}	{{1.7675, 20.}, {1.91891, 20.}}	{15.7435, 193.047}	inf	843.	1465.8	{1371.77, 1557.76}	73.8792	0.239542	newly	835.	511.38	{481., 543.}	38.7569	0.2135	Reff	0.933487	1.01382	{0.955715, 1.07025}	8.61183	ar	6528.	9179.23	{8846., 9444.}	40.6132
43	R0	{1.56986, 5.44652}	{2.6214, 18.7562}	{{2.32909, 15.7236}, {2.81462, 20.}}	{66.9829, 244.371}	inf	706.	2838.77	{2404.03, 3181.07}	302.092	0.601283	newly	846.	1065.47	{979., 1128.}	25.9823	0.0994328	Reff	0.905652	1.10351	{0.988745, 1.21536}	21.8464	ar	6312.	10 396.4	{10 041., 10 648.}	64.709
44	R0	{1.47523, 8.07785}	{1.85885, 19.3436}	{{1.59621, 15.207}, {2.00337, 20.}}	{26.0039, 139.465}	inf	601.	1458.86	{1000.14, 1702.41}	142.739	0.378891	newly	507.	529.37	{459., 607.}	9.86785	0.0414021	Reff	0.965243	1.07	{1.01357, 1.15106}	10.8847	ar	5692.	8850.62	{7915., 9280.}	55.4923
45	R0	{1.6417, 7.69429}	{2.23603, 18.9275}	{{2.08891, 17.3418}, {2.3975, 20.}}	{36.2023, 145.994}	inf	911.	2260.01	{2029.13, 2563.47}	148.08	0.391546	newly	777.	826.14	{755., 880.}	7.79151	0.0324524	Reff	0.893577	1.01623	{0.92742, 1.08818}	13.9091	ar	6735.	9963.35	{9657., 10 337.}	47.9339
46	R0	{1.38865, 5.83473}	{1.85343, 18.387}	{{1.63738, 14.8712}, {2.12284, 20.}}	{33.4886, 215.13}	inf	383.	1328.8	{897.542, 1588.37}	246.944	0.530009	newly	442.	509.92	{419., 578.}	17.5792	0.0692745	Reff	0.981081	1.16531	{1.06769, 1.27791}	18.845	ar	5233.	8571.97	{7729., 9220.}	63.806
47	R0	{1.45289, 6.87903}	{1.77643, 16.8104}	{{1.53403, 11.423}, {2.1231, 20.}}	{22.4035, 144.372}	inf	477.	1185.37	{717.984, 1634.36}	148.505	0.377043	newly	487.	493.48	{413., 583.}	10.4312	0.0447505	Reff	1.01252	1.12754	{0.982986, 1.2243}	12.2891	ar	5379.	8000.17	{6890., 9081.}	48.7297
48	R0	{1.69416, 9.4831}	{1.95137, 20.}	{{1.86636, 20.}, {2.12095, 20.}}	{15.1824, 110.901}	inf	944.	1601.66	{1480.52, 1713.58}	69.6676	0.228946	newly	702.	561.85	{513., 600.}	19.9644	0.0975315	Reff	1.03412	1.06519	{0.998341, 1.14416}	4.97929	ar	6925.	9092.94	{8797., 9407.}	31.306
49	R0	{1.47843, 7.16286}	{1.96749, 19.0039}	{{1.75562, 16.1922}, {2.16192, 20.}}	{33.0794, 165.311}	inf	577.	1538.47	{1177.69, 1832.24}	166.633	0.420119	newly	548.	569.93	{493., 663.}	9.27555	0.0392225	Reff	0.972659	1.14017	{1.0318, 1.20773}	17.2221	ar	5449.	8897.44	{8339., 9379.}	63.2857
50	R0	{1.68843, 5.72427}	{2.7681, 18.1825}	{{2.34587, 14.5801}, {3., 20.}}	{63.9451, 217.638}	inf	853.	2997.71	{2405.08, 3430.29}	251.432	0.538263	newly	913.	1110.29	{1023., 1182.}	21.7601	0.0847876	Reff	0.853333	0.895324	{0.806671, 0.967776}	7.13247	ar	6800.	10 179.8	{9806., 10 498.}	49.7029
